# Loss of transglutaminase 2 sensitizes for diet-induced obesity-related inflammation and insulin resistance due to enhanced macrophage c-Src signaling

**DOI:** 10.1038/s41419-019-1677-z

**Published:** 2019-06-05

**Authors:** Tibor Sághy, Krisztina Köröskényi, Krisztina Hegedűs, Miklós Antal, Csaba Bankó, Zsolt Bacsó, Attila Papp, Rinke Stienstra, Zsuzsa Szondy

**Affiliations:** 1 Dental Biochemistry, Faculty of Dentistry, University of Debrecen, Debrecen, Hungary; 2 Department of Biochemistry and Molecular Biology, Faculty of General Medicine, University of Debrecen, Debrecen, Hungary; 3 Department of Anatomy, Histology and Embryology, Faculty of General Medicine, University of Debrecen, Debrecen, Hungary; 4 Department of Biophysics and Cell Biology, Faculty of General Medicine, University of Debrecen, Debrecen, Hungary; 5 Division of Human Nutrition and Health, Wageningen University, Wageningen, The Netherlands; 6 Department of Internal Medicine, RadboudUMC, Nijmegen, The Netherlands

**Keywords:** Apoptosis, Obesity

## Abstract

Transglutaminase 2 (TG2) is a multifunctional protein that promotes clearance of apoptotic cells (efferocytosis) acting as integrin β_3_ coreceptor. Accumulating evidence indicates that defective efferocytosis contributes to the development of chronic inflammatory diseases. Obesity is characterized by the accumulation of dead adipocytes and inflammatory macrophages in the adipose tissue leading to obesity-related metabolic syndrome. Here, we report that loss of TG2 from bone marrow-derived cells sensitizes for high fat diet (HFD)-induced pathologies. We find that metabolically activated TG2 null macrophages express more phospho-Src and integrin β_3_, unexpectedly clear dying adipocytes more efficiently via lysosomal exocytosis, but produce more pro-inflammatory cytokines than the wild type ones. Anti-inflammatory treatment with an LXR agonist reverts the HFD-induced phenotype in mice lacking TG2 in bone marrow-derived cells with less hepatic steatosis than in wild type mice proving enhanced lipid clearance. Thus it is interesting to speculate whether LXR agonist treatment together with enhancing lysosomal exocytosis could be a beneficial therapeutic strategy in obesity.

## Introduction

Transglutaminase 2 (TG2) is a unique member of the transglutaminase family with various biological functions^1^. Although TG2 is localized predominantly within the cell, substantial amounts of the protein is present also on the surface of macrophages functioning as an integrin β_3_ coreceptor^2,3^. Acting so, TG2 in macrophages promotes phagocytosis of apoptotic cells by stabilizing the interaction between α_v_β_3_ integrins and the bridging molecule milk fat globule-EGF-factor 8 (MFG-E8)^4,5^. Increasing evidence indicate that impaired clearance of apoptotic cells is associated with the development of various chronic inflammatory diseases^6^. This is related to the fact that (1) uncleared apoptotic cells undergo secondary necrosis and induce inflammation, and (2) macrophages properly engulfing apoptotic cells would normally induce anti-inflammatory mechanisms that prevent tissue inflammation. In accordance, in TG2 null mice following in vivo apoptosis induction, apoptotic cells are not only accumulated in tissues, but are also surrounded by mononuclear cells^7^, and their clearance is accompanied by production of pro-inflammatory cytokines^8^. In addition, TG2 knockout mice develop systemic lupus erythematosus-like autoimmunity on long term^7^.

Multiple lines of evidence revealed that obesity is also characterized by low-grade inflammation that originates from the expanding adipose tissue^9^. Inflammation, predominantly in the visceral fat tissue is believed to be initiated and maintained by BH3 interacting domain death agonist (Bid)-mediated apoptosis of hypertrophic lipid overloaded adipocytes^10^ releasing chemoattractants, such as monocyte chemoattractant protein-1 (MCP-1)^11^ to recruit macrophages for their proper clearance. While adipose tissue macrophages in lean mice have an anti-inflammatory phenotype, recruited macrophages exposed to high concentrations of lipids switch their phenotype to the so-called metabolically activated one (MMe) characterized by high rate of lysosomal biogenesis, expression of CD11c and release of pro-inflammatory cytokines and resistin^12,13^. Since the size of adipocytes greatly excess that of the recruited macrophages, instead of being phagocytosed by a single macrophage, dead adipocytes are cleared by groups of recruited macrophages. It was estimated that >90% of macrophages infiltrating the adipose tissue of obese animals and humans are arranged around the dead adipocytes forming characteristic crown-like structures (CLS)^14^. During this process macrophages form a tight attachment on the dead adipocyte, acidify the contact space by activating the plasma membrane proton pumping V-ATPase and release their lysosomal content through lysosomal exocytosis machinery^15^. Initiation of both the lysosomal exocytosis program and the pro-inflammatory cytokine formation in metabolically activated macrophages seem to involve TLR2, while TLR4 activation contributes to the pro-inflammatory cytokine formation^16,17^.

Lipid-loaded adipocytes produce pro-inflammatory cytokines themselves, and the production is positively correlated with the degree of hypertrophy^18^. Lipid accumulation also alters adipocyte adipokine synthesis leading to enhanced release of leptin and resistin, and to decreased production of adiponectin^19^. Since adiponectin increases, while resistin and pro-inflammatory cytokines decrease insulin sensitivity, altogether these changes in the cytokine and adipokine expressions decrease whole-body insulin sensitivity leading to compensatory increases in circulating plasma insulin levels and later to the development of type-2 diabetes mellitus^20–23^.

In addition, macrophage-derived TNF-α^24,25^ and adipocyte-derived leptin^26^ also contribute to the cell death induction of hypertrophic adipocytes. As a result, during long term obesity more and more hypertrophic adipocytes die leading to a marked loss of the visceral adipose tissue size and to relocation of its lipid depots to other tissues, such as liver, where the lipid accumulation promoted by TNFα-mediated upregulation of hepatic fatty acid translocase leads to hepatic steatosis^27,28^.

In the present paper, we investigated whether TG2 is involved in the clearance of apoptotic adipocytes, and how its loss affects obesity-related gonadal fat adipocyte apoptosis, macrophage recruitment, inflammation, insulin resistance, and hepatic steatosis during diet-induced obesity in mice.

## Results

### Loss of TG2 does not affect weight gain during diet-induced obesity, but enhances adipose tissue inflammation

**Fig. 1:**
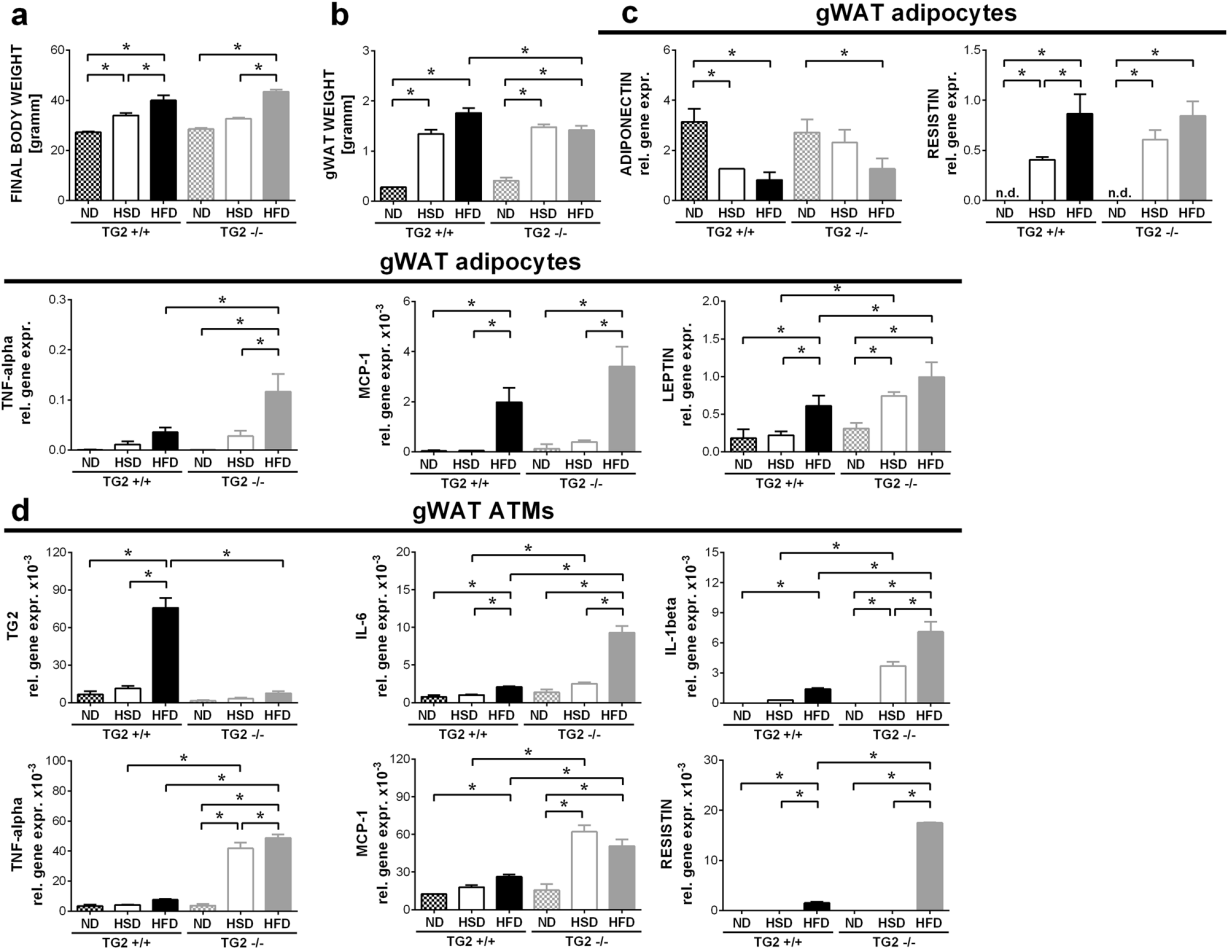
Loss of TG2 does not affect weight gain during diet-induced obesity, but enhances the pro-inflammatory cytokine production by the gonadal white adipocytes and adipose tissue macrophages. **a** Body weights of wild type and TG2 null mice fed on normal (ND), high-sucrose/low-fat (HSD) or high-sucrose/high-fat (HFD) diet at the end of the feeding period. **b** Gonadal fat weights of wild type and TG2 null mice fed on ND, HSD or HFD at the end of the feeding period. **c** Adiponectin, resistin, TNF-α, MCP-1, and leptin relative gene expression levels of gWAT adipocytes in wild type and TG2 null mice fed on ND, HSD or HFD determined by qRT-PCR at the end of the feeding period. GAPDH was used as reference gene. **d** TG2, IL-1β, IL-6, TNF-α, MCP-1, and resistin relative gene expression levels of gWAT macrophages in wild type and TG2 null mice fed on ND, HSD or HFD determined by qRT-PCR at the end of the feeding period. GAPDH was used as a reference gene. Results are expressed as mean ± SD (*n* = 8 mice per group). Statistical significance was evaluated by one-way ANOVA test (*p < 0.05)

Wild type mice exposed to either high sucrose/high fat diet (HFD) or to high sucrose diet (HSD) developed marked obesity compared with animals kept on a standard control diet (ND) (40.0 ± 5.5 versus 33.9 ± 2.6 versus 27.2 ± 0.6 g body weight of mice kept on HFD, HSD or ND, respectively, *p* < 0.01). Loss of TG2 did not affect significantly the weight gain of the animals kept on the three different types of diet (Fig. 1a).

As seen in Fig. 1b, the gonadal fat tissue (gWAT) weight also increased significantly in both wild type and TG2 null mice exposed to HSD or HFD as compared to mice kept on control diet. However, in case of TG2 null mice kept on HFD the gonadal fat was significantly less than in wild type mice kept on HFD very likely due to the enhanced adipocyte apoptosis in TG2 null gonadal fat at this time point (see later).

Loss of TG2 did not affect the obesity-related alterations in the expression levels of the adiponectin and resistin (Fig. 1c), but obesity-induced expressions of TNF-α, MCP-1, and leptin were significantly enhanced in the gWAT adipocytes of TG2 null animals as compared to wild type mice (Fig. 1c).

HSD and HFD significantly induced the mRNA expression of the pro-inflammatory cytokines in adipose tissue macrophages as well, which was more pronounced in the absence of TG2 (Fig. 1d). All these data indicate that though loss of TG2 does not affect weight gain, inflammation is much more pronounced in the gWAT of TG2 null animals during diet-induced obesity.

### Enhanced adipocyte apoptosis in the gonadal fat during diet-induced obesity in TG2^−/−^ mice

As obesity promotes adipocyte death, we decided to determine the expression of various pro- and anti-apoptotic genes in the adipocytes of gWAT. As seen in Supplementary Fig. S1, in the gWAT adipocytes not only the expression of the pro-apoptotic Bid, but the expression of the pro-apoptotic Bim was also induced during diet-induced obesity.

Interestingly, not only the pro-apoptotic, but mRNAs of two anti-apoptotic genes, Bcl-2 and Bcl-xl (Supplementary Fig. S1c, d), were also induced during diet-induced obesity. But the levels of Bcl-2 mRNA were significantly dropped in the TG2 null mice kept on HFD as compared to their wild type counterparts. On the other hand, the anti-apoptotic Mcl-1 levels were decreased (Supplementary Fig. S1e), while the expression of TG2 was not altered in the gWAT adipocytes during diet-induced obesity (Supplementary Fig. S1f).

Altogether these data are indicative for more diet-induced adipocyte death in the gWAT of mice kept on HFD in the absence of TG2. Indeed, in the gWAT of TG2 null mice kept on either HSD or HFD significantly more dying cells were detected than in their wild type counterparts (Supplementary Fig. S1g) by identifying dead adipocytes as labeled by CLS^16,29^.

### Loss of TG2 enhances hepatic steatosis and insulin resistance in mice during diet-induced obesity

Since increased gonadal fat apoptosis and high pro-inflammatory cytokine production are associated with ectopic fat storage including hepatic steatosis^10^ and insulin resistance^16^, we measured the development of hepatic steatosis, alterations in glucose tolerance and in insulin resistance in these mice. Together with the enhanced gWAT adipocyte apoptosis (Fig. 1b and Supplementary Fig. S1), in TG2 null mice kept on HFD both the liver weight (Supplementary Fig. S2a) and the triacylglycerol content of the livers (Supplementary Fig. S2b) were significantly higher than that of the wild type mice kept on HFD indicating translocation of more lipids from the gonadal fat into the liver. Tissue sections of livers stained with haematoxylin eosin confirmed these findings (Supplementary Fig. S2c).

Glucose tolerance tests demonstrated that as compared to mice fed on ND, HSD and HFD decreases the glucose tolerance. However, loss of TG2 did not worsen the results of the glucose tolerance tests (Supplementary Fig. S2d). Based on insulin tolerance tests, however, animals lacking TG2 fed on HFD are more insulin resistant (Supplementary Fig. S2e). These results were corroborated by enhanced fasting plasma insulin levels as compared to their wild type counterparts (Supplementary Fig. S2f). Altogether these data indicate that similar to SLE^7^ or atherosclerosis^30^, loss of TG2 sensitizes also for the development of obesity-induced inflammation and that of its consequences.

### Loss of TG2 in non-bone marrow-related cells leads to enhanced circulating plasma insulin levels and insulin resistance

TG2 is expressed not only by macrophages, but also by adipocytes^31^ and hepatocytes^32^. To determine, whether loss of TG2 in bone marrow-derived cells or in other cell types is responsible for the above changes, wild type or TG2 null mice were terminally irradiated, and their bone marrow was replaced by a bone marrow originated from either wild type or TG2 null mice in each combination. To confirm successful ablation and reconstitution, we involved wild type BoyJ (a CD57BL/6 variant strain) mice in these experiments, which express the CD45.1 allelic variant of CD45, while C57BL/6 mice are CD45.2 positive. Hematologic and flow cytometric analysis of these mice following bone marrow transplantation demonstrated a leukocyte repopulation over 95%.

Irradiated CD57BL/6 mice gained significantly less weight, than their non-irradiated counterparts by the end of the 16 weeks feeding (Supplementary Fig. S3a), in line with previously published studies^33,34^. As compared to the wild type counterparts, loss of TG2 in the non-bone marrow-derived cells did not affect significantly the percentage of dying adipocytes, the gonadal weight, gWAT adipocyte Bid expression, liver weight, liver triacylglycerol content or adipocyte pro-inflammatory cytokine or adipokine mRNA expression either, with the exception of TNF-α (Supplementary Fig. S3b–h). On the other hand, the amount of resistin and IL-6 produced by adipose tissue macrophages were significantly higher in mice lacking TG2 in in the non-bone marrow-derived cells with no alterations in their TNF-α or MCP-1 production (Supplementary Fig. S3i). In addition, we could detect significantly increased fasting circulating plasma insulin levels and increased insulin resistance by using insulin tolerance test (Supplementary Fig. S3i, k). Altogether our data indicate that loss of TG2 in non-bone marrow-derived cells induces early insulin resistance without significantly affecting the development of adipocyte cell death, inflammation and hepatic steatosis induced by HFD.

### Loss of TG2 from bone marrow-derived cells is responsible for the obesity-related adipocyte cell death, inflammation and hepatic steatosis in TG2 null mice

**Fig. 2:**
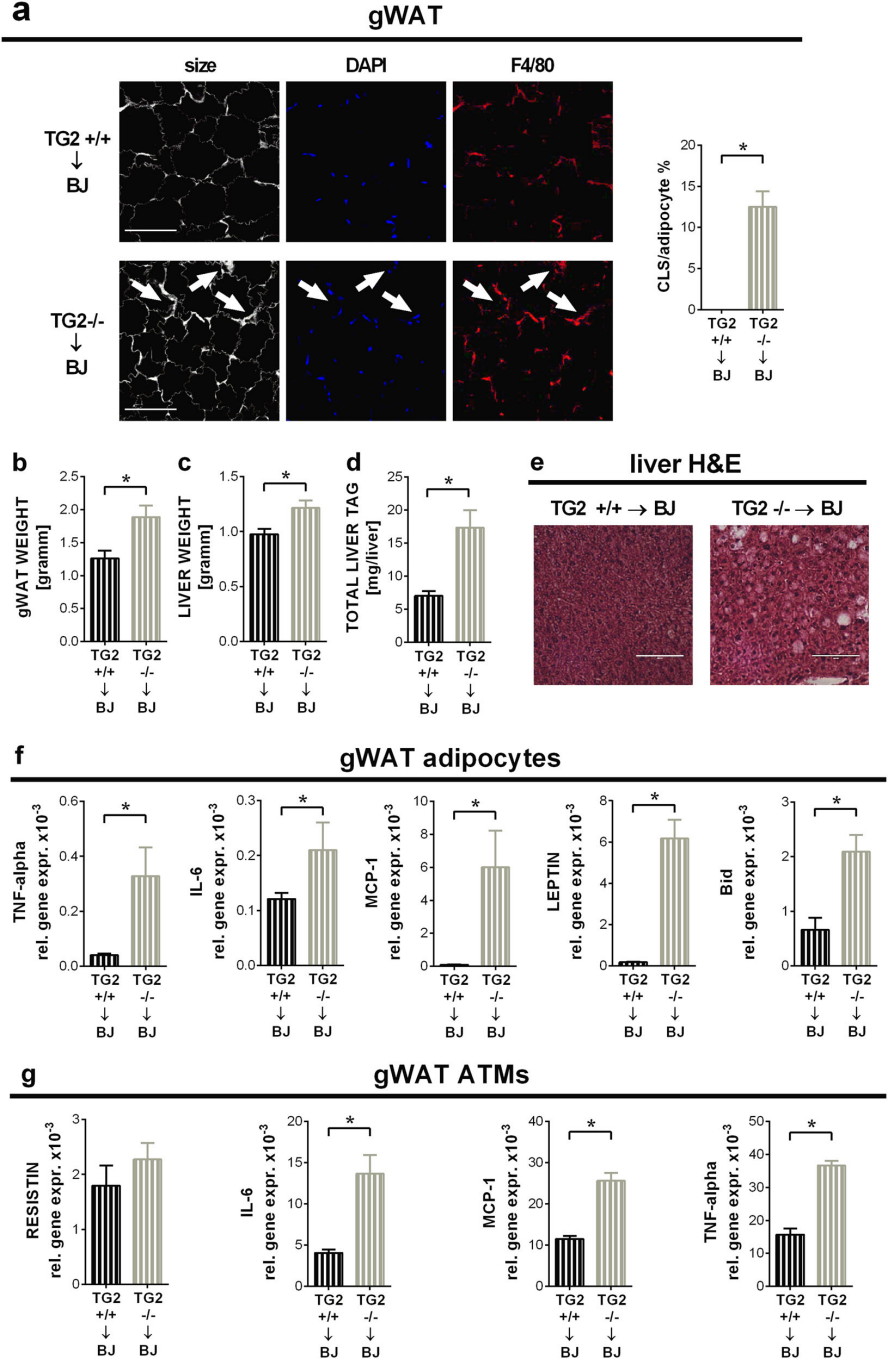
Loss of TG2 in BMD cells promotes adipocyte cell death, inflammation and hepatic steatosis in mice fed on HFD. **a** Confocal images of gWAT collected from BoyJ mice transplanted with the bone marrow of either TG2^+/+^ or TG2^−/−^ mice at the end of the HFD feeding period. Paraffin-embedded gWAT slides were stained with the non-specifically labeling anti-digoxin antibody, anti-F4/80 antibody and DAPI to visualize adipocytes, macrophages, and nuclei under confocal microscopy. Scale bar, 100 μm. CLS cells in fields from randomly selected sections of three different mice in each group were quantified. Results are expressed as mean ± SD (*n* = 3 mice per group). Statistical significance was evaluated by 2-tailed unpaired Student’s *t*-test (*p < 0.05). **b** Weights of gWAT from the mice described in (**a**). **c** Liver weights from the same mice. **d** Liver triacylglycerol contents from the same mice determined from saponified, neutralized liver extracts by glycerol enzymatic assay. **e** Paraffin-embedded liver tissue slides from the same mice stained with H&E to visualize tissue architecture. One representative series of three are shown. Scale bar, 250 μm. **f** Inflammatory cytokine, leptin and Bid relative gene expression levels of gWAT adipocytes from the same mice determined by qRT-PCR using GAPDH as a reference gene. **g** Inflammatory cytokine and resistin relative gene expression levels of gWAT macrophages from the same mice determined by qRT-PCR using GAPDH as a reference gene. Results are expressed as mean ± SD (*n* = 8 mice per group). Statistical significance was evaluated by one-way ANOVA (*p < 0.05)

Absence of TG2 in bone marrow-derived cells, however, promoted adipocyte death in the gWAT of mice exposed to HFD, compared to mice transplanted with wild type bone marrow (Fig. 2a), possibly due to the enhanced TNF-α^24,25^and leptin^26^ production in these mice. In addition, significantly larger gonadal fat weight (Fig. 2b), increased liver weight and TAG content (Fig. 2c, d) could be detected in animals lacking TG2 in the bone marrow compartment. Increased hepatic steatosis was confirmed by haematoxylin eosin stained liver sections as well (Fig. 2e). In line with these data, in the absence of bone marrow-derived cells' TG2 a significantly higher expression of adipose tissue TNF-α, IL-6, MCP-1, leptin and Bid mRNA-s were detected in mice exposed to HFD (Fig. 2f). Similarly, gWAT macrophage IL-6, MCP-1, TNFα mRNA levels were also significantly higher in the absence of TG2 (Fig. 2g). As shown later in Fig. 5j, circulating plasma levels were also higher in the absence of bone marrow-derived cells' TG2. Taken together, our data indicate that as compared to wild type mice, loss of TG2 from bone marrow-derived cells drives in TG2 null mice the enhanced inflammation and the more severe pathological consequences of HFD-feeding.

### Loss of TG2 in macrophages results in a more efficient lysosomal exocytosis

**Fig. 3:**
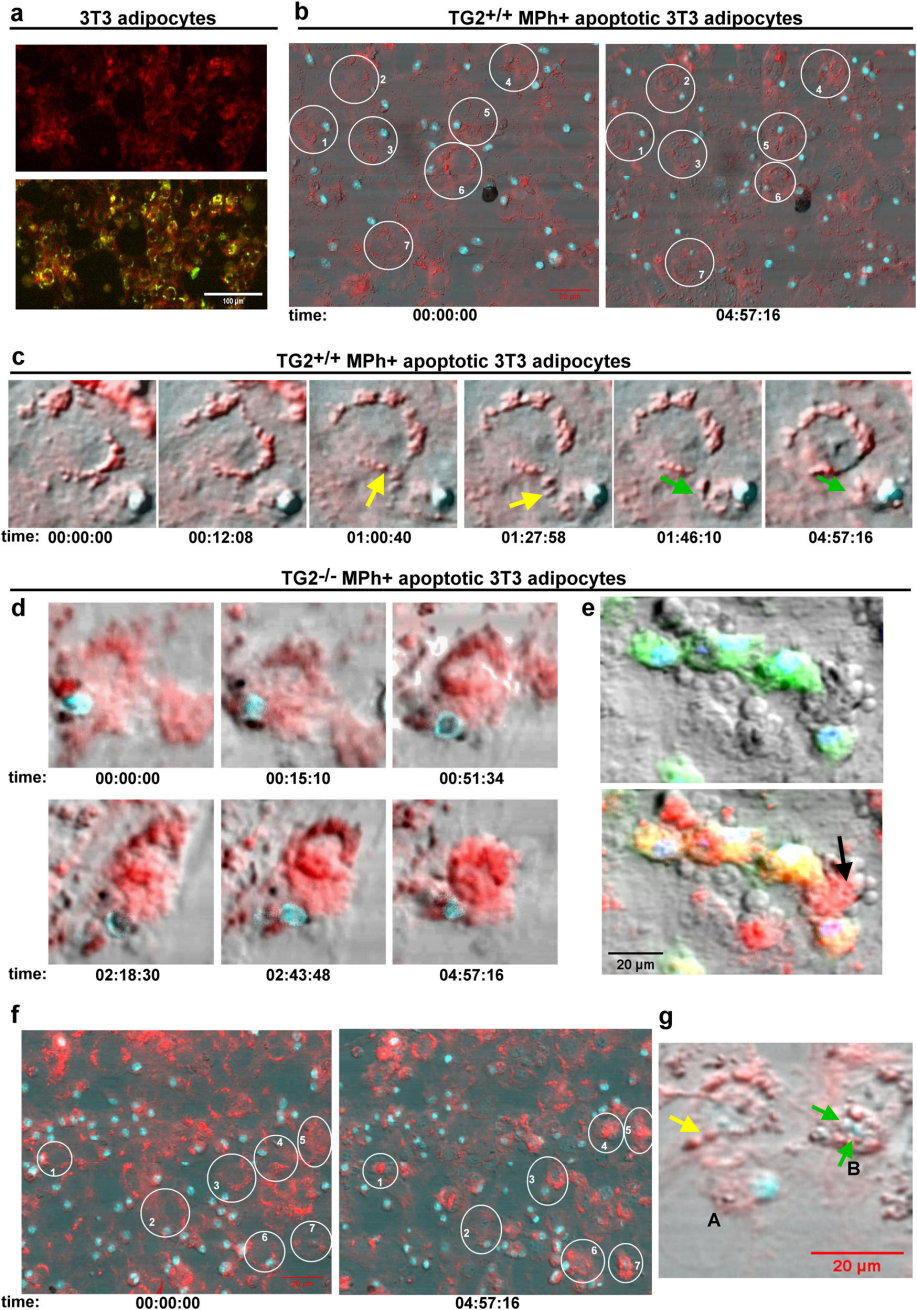
Clearance of apoptotic adipocytes is faster by TG2 null macrophages than by their wild type counterparts. **a** Apoptotic adipocytes form lipid-containing vesicles surrounded by phosphatidylserine positive membrane on their cell surface. Laser scanning cytometric image of apoptotic 3T3 adipocytes induced to die by serum withdrawal for 24 h and stained for lipids with Nile red (red) and for phosphatidylserine with FITC-labeled Annexin V (green). Scale bar, 100 μm. **b** 3T3 adipocytes induced to die by serum withdrawal for 24 h were stained for lipids with Nile red (red) and exposed to wild type BMDMs stained with vital Hoechst DNA dye for nuclei (blue). Apoptotic adipocyte clearance was followed for 5 h by laser scanning microscopy. First and last images from the Supplementary video 2 demonstrating the state of clearance for several apoptotic adipocyte/macrophage cell pairs at the end of the 5 h period. Please, note that apoptotic adipocytes shrink very slow (adipocyte 4 and 6) or not yet in the presence of wild type macrophages. **c** One apoptotic adipocyte and one wild type macrophage pair (from the Supplementary video 1) demonstrating the process of lysosomal exocytosis. 3T3 adipocytes induced to die by serum withdrawal for 24 h were stained for lipids with Nile red and exposed to wild type BMDMs stained with vital Hoechst DNA dye for nuclei (blue). Apoptotic adipocyte clearance was followed for 5 h by laser scanning microscopy. Yellow arrows indicate lipid-containing vesicle uptake by macrophages, green arrows point to vesicles already in the macrophage. The fact that the cytosol of macrophage contains red vesicles indicates lipid uptake from the adipocyte. **d** One apoptotic adipocyte and one TG2 null macrophage pair (from the video 3) demonstrating the process of the apoptotic adipocyte uptake by TG2 null macrophages. 3T3 adipocytes induced to die by serum withdrawal for 24 h were stained for lipids with Nile red and exposed to TG2 null BMDMs stained with vital Hoechst DNA dye for nuclei (blue). Apoptotic adipocyte clearance was followed for 5 h by laser scanning microscopy. Due to the fast vesicle uptake, the cytosol of the TG2 null macrophage is full with red vesicles making difficult to see the border between the adipocyte and the macrophage. Please, note apoptotic adipocyte cell membrane blebbing and the shrinkage of the adipocyte on the last image. **e** To visualize macrophages and adipocytes better, this is a laser scanning cytometric image of apoptotic 3T3 adipocytes induced to die by serum withdrawal for 24 h stained for lipids with Nile red and being engulfed by TG2 null BMDMs stained with vital Hoechst DNA dye for nuclei (blue) and CFDA for cytosol (green) after 4 h of phagocytosis. Upper panel shows the image without the red color of fat, lower panel shows all the colors together. Please note, that the green cytosol of BMDMs becomes yellow due to lipid uptake as the red and green colors overlap. Arrow indicates a shrinked blebbing adipocyte. Scale bar 20 μm. **f** 3T3 adipocytes induced to die by serum withdrawal for 24 h were stained for lipids with Nile red and exposed to TG2 null BMDMs stained with vital Hoechst DNA dye for nuclei (blue). Apoptotic adipocyte clearance was followed for 5 h by laser scanning microscopy. First and large images from the Supplementary video 4 demonstrating the state of clearance for several apoptotic adipocyte/macrophage cell pairs at the end of the 5 h period. Please, note that all the labeled adipocytes shrink and bleb. **g** After completing lysosomal exocytosis TG2 null macrophages engulf adipocyte nuclei. Laser scanning cytometric image of 3T3 adipocytes induced to die by serum withdrawal for 24 h stained for lipids with Nile red and exposed to TG2 null BMDMs stained with vital Hoechst DNA dye for nuclei (blue). At this stage of adipocyte clearance, the Hoechst slightly stains the adipocyte nucleus as well. Yellow arrow indicates the last lipid-containing vesicles’ uptake by macrophage A. Green arrows indicate nuclei taken up by macrophage B. Scale bar, 20 μm

Since our initial hypothesis was that the enhanced obesity-related inflammation in TG2 null mice is the result of impaired apoptotic cell phagocytosis, we decided to determine whether macrophage TG2 is required for the clearance of apoptotic adipocytes. For this purpose, adipocytes differentiated from 3T3 fibroblasts were made apoptotic using serum deprivation, exposed to both wild type and TG2 null bone marrow-derived macrophages and their clearance as a function of time was followed by laser scanning cytometry. Surprisingly, as shown in Fig. 3 and Supplementary Video 1–4, loss of macrophage TG2 did not delay, but accelerated lysosomal exocytosis of dead adipocytes. We have revealed that adipocytes entering apoptosis form first lipid-containing vesicles surrounded by phosphatidylserine positive membranes indicative of cell death (Fig. 3a). Macrophages attach to the dead adipocyte as it was described^15^, and continuously engulf these lipid-containing vesicles. One wild type macrophage acting on one dead adipocyte keeps taking up these vesicles during the whole 5 h time frame of the video (Fig. 3b, c, Supplementary Video 1 and 2), but does not complete the clearing. TG2 null macrophages, however, take up these lipid-containing vesicles (Fig. 3d–f, Supplementary Video 2 and 3), and trigger the initiation of the classical apoptosis program characterized by membrane blebbing much faster. After the whole cytosolic content of the adipocyte is cleared, TG2 null macrophages digest finally the degrading nucleus (Fig. 3g). None of the wild type adipocytes could reach this final engulfment stage during the video time frame (Supplementary Video 2).

### Metabolically activated TG2 null macrophages produce more pro-inflammatory cytokines than their wild type counterparts due to enhanced c-Src signaling

**Fig. 4:**
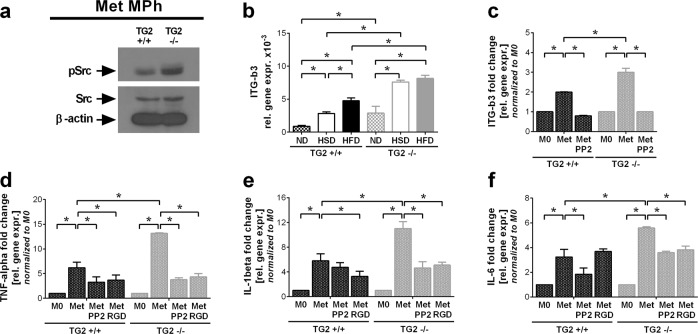
Metabolically activated TG2 null macrophages produce more pro-inflammatory cytokines than their wild type counterparts due to enhanced c-Src signaling. **a** c-Src and p-Src levels of wild type and TG2 null metabolically activated BMDMs determined by Western blot analysis using β-actin as reference protein. **b** Relative gene expression levels of integrin β_3_ in gWAT macrophages from wild type and TG2 null mice kept on ND, HSD or HFD at the end of the feeding period determined by qRT-PCR using GAPDH as a reference gene. **c**–**f** Relative gene expression levels of integrin β_3_, TNF-α, IL-1β, and IL-6 in metabolically activated wild type or TG2 null BMDMs alone or in the presence of 2 μM PP2 or 0.5 mg/ml RGD peptide determined by qRT-PCR using GAPDH as a reference gene. Results are expressed as mean ± SD (*n* = 8 mice per group, *n* = 4 for metabolically activated macrophages). Statistical significance was evaluated by two-way ANOVA (*p < 0.05)

Previous studies have demonstrated that in the absence of TG2 integrin β_3_ signaling is altered leading to enhanced c-Src tyrosine kinase activity^35^. Accordingly, in TG2 null macrophages the amount of phosphorylated c-Src, β_3_ integrin levels and c-Src-dependent integrin β_3_ phosphorylation are enhanced leading to decreased basal IκB levels, and consequently to enhanced pro-inflammatory cytokine transcription following exposure to lipopolysaccharide^36^. Similar to lipopolysaccharide, palmitate was also shown to activate TLR4^17^ and c-Src^37^. Thus we determined the pSrc levels in MMe macrophages that we generated in vitro as it was previously described^16^, and found increased levels in the absence of TG2 (Fig. 4a). Next we determined the mRNA levels of integrin β_3_ in gWAT macrophages and found that its levels are increased during HSD and HFD, and both the basal and the HSD/HFD-induced levels are significantly higher in the absence of TG2 (Fig. 4b). Similarly, in the absence of TG2 we detected a significantly increased expression of integrin β_3_ in MMe macrophages as well (Fig. 4c). Induction of integrin β_3_ expression in MMe macrophages was completely c-Src-dependent, as PP2, a Src inhibitor, prevented it.

Metabolic activation also induced significantly higher TNF-α, IL-1β, and IL-6 mRNA levels in TG2 null MMe macrophages than in the wild type ones (Fig. 4d–f). In addition, the PP2 Src inhibitor and the integrin receptor inhibitor RGD peptide inhibited the induction of pro-inflammatory cytokine formation both in wild type and in TG2 null MMe macrophages, and in the presence of these inhibitors no difference in their metabolic activation-induced pro-inflammatory cytokine induction was found. These data confirm that TG2 null macrophages are more sensitive to inflammatory stimulation, and this is related to an enhanced integrin β3/ c-Src signaling.

### LXR agonist treatment reverts the HFD-induced phenotype in mice lacking TG2 in bone marrow-derived cells with less hepatic steatosis than in wild type mice

Liver X receptors (LXR) are nuclear receptors that play a key role in regulating whole-body cholesterol, fatty acid and glucose metabolism^38^. Activation of LXR receptors is also known to strongly suppress macrophage inflammation^39^. Though LXR agonist treatment has been shown to affect metabolism significantly during diet-induced obesity resulting in attenuated weight gain, induction of white adipocyte lipolysis and fatty acid oxidation^40^, hepatic steatosis^41^ and improved insulin sensitivity^42,43^, we reasoned, if loss of TG2 in macrophages affects primarily inflammation, LXR agonist treatment will result in a similar phenotype in mice carrying and in mice lacking TG2 in bone marrow-derived cells exposed to HFD.

**Fig. 5:**
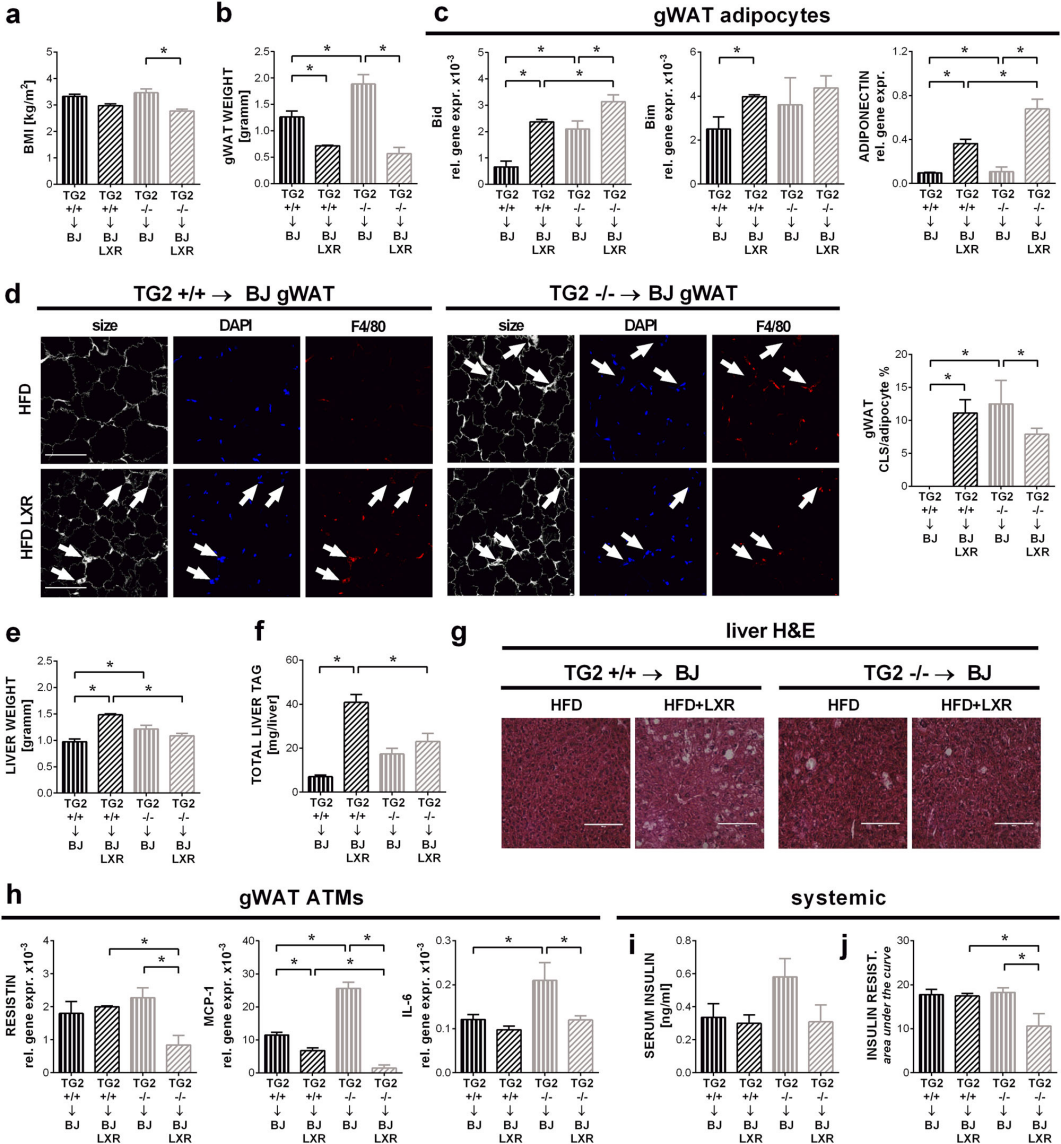
LXR agonist treatment reverts HFD-induced obesity with less hepatic steatosis in mice lacking TG2 in the bone marrow- derived cells. **a** BMI of BoyJ mice transplanted with the bone marrow of either TG2^+/+^ or TG2^−/−^ mice at the end of the 16-week HFD feeding period combined or not with the LXR agonist GW3965 (20 mg/kg/day) treatment. **b** gWAT weights of the same mice. **c** Relative gene expressions of Bid, Bim, and adiponectin of gWAT adipocytes from the same mice determined by qRT-PCR using GAPDH as a reference gene. **d** Confocal images of gWAT collected from the same mice. Paraffin-embedded gWAT slides were stained with the non-specifically labeling anti-digoxin antibody, anti-F4/80 antibody and DAPI to visualize adipocytes, macrophages, and nuclei under confocal microscopy. Scale bar, 100 μm. CLS cells in fields from randomly selected sections of three different mice in each group were quantified. Results are expressed as mean ± SD (*n* = 3 mice per group). Statistical significance was evaluated by 2-tailed unpaired Student’s *t*-test (*p < 0.05). **e** Liver weights of the same mice. **f** Liver triacylglycerol contents from the same mice determined from saponified, neutralized liver extracts by glycerol enzymatic assay. **g** Paraffin-embedded liver tissue slides from the same mice stained with H&E to visualize tissue architecture. One representative series of three are shown. Scale bar, 250 μm. **h** MCP-1, IL-6, and resistin relative gene expression levels of gWAT macrophages from the same mice determined by qRT-PCR using GAPDH as a reference gene. **i** Serum insulin levels determined by Mouse Insulin ELISA kit. **j** Insulin resistance values of the same mice. Insulin resistance test was performed on week 15 (6 h fasting followed by intraperitoneal administration of 0.75 IU/bwkg insulin). Data are presented as mean ± SD (*n* = 8 mice per group). Statistical significance was evaluated by two-way ANOVA (*p < 0.05)

Thus, we exposed bone marrow transplanted mice expressing or not TG2 in their bone marrow-derived cells to the LXR agonist GW3965 mixed with their high fat containing food during the whole feeding period, as described^44^. In agreement with a previous publication^42^, LXR agonist treatment prevented body (Fig. 5a) and gonadal fat weight (Fig. 5b) gain in HFD-exposed mice. This was related to an enhanced white adipocyte apoptosis assessed by the enhanced Bid and Bim expression of gWAT adipocytes (Fig. 5c). In contrast, the percentage of dying adipocytes observed on tissue sections decreased in the gonadal fat of LXR-treated mice, if TG2 was missing from the bone marrow-derived cells (Fig. 5d), perhaps due to their more efficient clearance. Similarly, significantly increased LXR activation-related hepatic steatosis was detected only in wild type mice (Fig. 5e–g). LXR agonist-treated TG2 null gWAT macrophages produced significantly less MCP-1 and resistin, than their wild type counterparts (Fig. 5h), while the gWAT adipocytes from the same mice significantly more adiponectin (Fig. 5c) reflecting an improved insulin sensitivity. Indeed, though there was no difference in the fasting circulating plasma insulin levels in the LXR agonist treated mice (Fig. 5i), animals lacking TG2 in bone marrow-derived cells, showed increased insulin sensitivity (Fig. 5j).

## Discussion

Previous studies have demonstrated that impaired clearance of apoptotic cells is associated with the development of various chronic inflammatory diseases^6^. Since TG2 has been shown to participate in the apoptotic cell clearance^4,7^, and obesity is associated with enhanced adipocyte apoptosis, we decided to investigate whether loss of TG2 affects diet-induced obesity-related pathologies. Data presented in this paper indicate that loss of TG2 indeed enhances obesity-related pathologies such as adipose tissue inflammation, adipocyte death, hepatic steatosis and insulin resistance in mice exposed to either HSD or to HFD, and all this is a result of the loss of TG2 from bone marrow-derived cells.

Since our hypothesis was that the enhanced inflammation in TG2 null mice is related to impaired apoptotic cell clearance, we tested whether TG2 was also required for the clearance of the large, lipid-rich apoptotic adipocytes. Since macrophages were reported to interact only with dead adipocytes and the process involved actin polymerization^15^, we expected that TG2 might be involved in the process by promoting possible integrin β_3_/apoptotic adipocyte interaction, as it does during the classical efferocytosis process^4^. To our surprise, however, we found that dead adipocyte clearance is more effective by TG2 null macrophages. Our results clearly demonstrate that the apoptosis program of adipocytes is initiated with packing the cytosolic content into lipid-rich extracellular vesicles covered by phosphatidylserine positive membrane, and the lysosomal exocytosis of macrophages is a process of a continuous uptake of these vesicles that, according to Haka et al., might occur directly into the lysosome^15^. It is interesting to note that while we were writing this paper, it was published that living adipocytes also release lipid-containing extracellular vesicles (exosomes) that are taken up by the surrounding macrophages and the exosomes contributes to the macrophage metabolic programming^45^.

Since TG2 null macrophages are much more efficient in lysosomal exocytosis, impaired clearance of dead adipocytes cannot explain the observed HFD-induced phenotype of TG2 null mice, though we cannot exclude that enhanced lipid uptake itself might be pro-inflammatory. However, we could demonstrate that metabolically activated TG2 null macrophages produce more pro-inflammatory cytokines due to their enhanced integrin β_3_/c-Src signaling^35,36^. Our observations are in line with other reports, which also demonstrated that α_v_β_3_ integrin signaling can induce NFκB activation in macrophages^46^, and that c-Src activity in macrophages is associated with various inflammatory responses^47^.

Interestingly, we found that both pro-inflammatory cytokine formation and lysosomal exocytosis, both TLR2-dependent^16^, were enhanced in TG2 null macrophages. TLR2 ligands are molecules with diacyl and triacylglycerol moieties^48^. Integrin β_3_ is part of the pre-formed TLR2/TLR1 signaling complex and is essential for triacyl lipopeptide engagement of TLR2 by recruiting triacyl lipopeptide bound to vitronectin^49^. In addition, α_v_β_3_-integrin signaling can boost the MYD88-dependent TLR2 signaling^50^. What is more, phosphatidylserine positive vesicles were already shown to be taken up by MFG-E8/integrin β_3_-dependent manner at least by dendritic cells^51^. Thus enhanced integrin β_3_ expression and signaling might be the explanation for the enhanced lysosomal exocytosis of TG2 null macrophages.

Finally, we also compared the response of mice to LXR agonist treatment during HFD-induced obesity with the expectation that after attenuation of adipose tissue inflammation the phenotype of mice carrying or not TG2 in their bone marrow-derived cells will be similar. LXR agonist treatment prevented HFD-induced obesity in both types of mice. In addition, as it was reported by others^42^, we also detected enhanced gWAT adipocyte apoptosis and increased hepatic steatosis in LXR agonist treated mice. Lack of gonadal weight gain as a result of LXR treatment was reported to be the consequence of the LXR agonist-induced activation of adipocyte LXRα which is known to promote lipolysis and fatty acid oxidation in adipose tissue^41^ leading finally to reduced adipocyte cell number. While, however, the rate of adipocyte apoptosis, based on the mRNA expression levels of Bid, was not higher in LXR agonist-treated wild type mice, their hepatic steatosis was significantly more pronounced than that of the LXR agonist-treated mice in which TG2 was absent from the bone marrow-derived cells. Previous studies indicated that the LXR agonist-induced hepatic steatosis is the result of activation of hepatocyte LXRα which triggers SREBP-c1-dependent triacylglycerol synthesis^41,52^. However, in our mice there was no difference in the genotype of the hepatocytes. Thus our observations indicate that TG2 null macrophages can better buffer metabolic tissues from the damage caused by ectopic accumulation of saturated free fatty acids^53^ very likely due to their enhanced lipid handling. Thus it is interesting to speculate whether LXR agonist treatment in combination with enhancing lysosomal exocytosis could be a therapeutic strategy in obesity.

## Materials and methods

### Animals and diets

Eight-week-old male TG2 deficient^54^ and wild type mice on a C57Bl/6 background generated from heterozygous parents were housed in separated cages (with water and food ad libitum) during the 16 weeks of feeding experiment. In the first 2 weeks, animals were kept on high-sucrose/low-fat (HSD; 10% kcal% fat; 17% kcal% sucrose; Research Diets Inc., D12450H) After this run-in period, mice were divided into two groups: the HSD group remained on HSD, whereas the HFD group received high-sucrose/high-fat (HFD; 45% kcal% fat, 17% kcal% sucrose; Research Diets Inc., D12451) diet during the following 14 weeks. The control group of mice were kept all the time on normal diet (ND; 13% kcal% fat; 4.6% kcal% sucrose; Special Diets Services, VRF1 (P)). Bone marrow transplanted (BMT) mice were maintained in a specific-pathogen-free status (autoclaved top filter cages) for the entire course of experimentation, and antibiotics (amoxicillin antibiotic, clavulanic acid, 500 mg/125 mg l−1 of drinking water) were administered in the drinking water following 4 weeks post-transplantation. Following 2 weeks of ND after BMT, they were kept on HFD diet for 16 weeks. For LXR ligand treatment in vivo, mice were fed after the bone marrow transplantation on HFD supplemented with 20 mg/kg/day GW3965 (AbMole, M1929) as described above. Mice were maintained under a 12 h light: 12 h darkness cycle and had access to food and water ad libitum. The body weight and food intake of the animals were registered weekly. For tissue collection, mice were killed by isoflurane overdose at week 17 in accordance to the University of Debrecen. Study protocols were approved by the Animal Care Committee of the University of Debrecen (DEMÁB).

### Bone marrow transplantation

Recipient BoyJ, TG2 null and TG2^+/+^ wild mice (7 weeks old, males) were irradiated with 11 Gy using a Theratron 780C cobalt unit for the ablation of the recipient bone marrow. The animals to be irradiated were immobilized using a circular cage (mouse pie cage) that could hold up to 11 mice (alert mice). Following irradiation, isolated bone marrow cells (in sterile RPMI-1640 medium) flushed out the femur, tibia, and humerus from donor BoyJ, TG2 null or TG2+/+ mice were transplanted into the recipient mice by retro-orbital injection (20 × 10^6^ bone marrow (BM) cells per mouse). This experimental BMT CD45 congenic model allowed us to detect donor, competitor and host contributions in hematopoiesis and repopulation efficiency of donor cells (congenic mice with CD45.1 versus CD45.2). The CD45.1 and CD45.2 contribution were then detected by flow cytometry usually 8–12 weeks following BMT. In short a cut at the tail tip of the mice provided a drop of blood that was placed into 0.5 ml phosphate-buffered saline (PBS) + 1% fetal bovine serum + 10 U ml^−1^ heparin buffer (Sigma Aldrich H3393) (samples kept on ice). The cells were directly stained by 2 μl mouse anti-mouse CD45.2-FITC (clone 104) and 2 μl rat anti-mouse GR1-PE (clone RB6-8C5) antibodies (BD Pharmingen) and incubated on ice for 30 min. After two washes with ice-cold PBS–fetal bovine serum–heparin buffer, cells were resuspended in 0.5–1 ml BD FACS lysing solution (BD cat no. 349202), incubated for 5 min at RT then centrifuged (400 g, 5 min, 4 °C). The double stained samples were run on FACS (BD FACS Calibur) and the ratio of donor cells was determined. The repopulation was over 95% gated on the granulocyte fraction.

### Isolation and metabolic activation of BMDMs

Bone marrow cells were isolated from femurs of 3–6 months old male TG2 null mice and their wild type counterparts. Bone marrow macrophages (BMDMs) were differentiated in DMEM supplemented with 10% FBS (12106C), 2 mM L-glutamine (G7513), 1 mM Na-pyruvate (S8636), 50 μM 2-mercaptoethanol (M3148) and 100 U/ml penicillin/100 μg/ml streptomycin (P4333) all from Sigma-Aldrich and 10% L929 fibroblast conditioned media for 7 days. For metabolic activation, differentiated macrophages were treated with a combination of 30 mM D-glucose (G8270), 10 nM insulin (12643) and 0.4 mM sodium-palmitate (P9767) all from Sigma-Aldrich for 24 h (16). Sodium palmitate was prepared by diluting a 200 mM stock solution in 70% ethanol into 10% fatty acid-free, low-endotoxin BSA (Sigma Aldrich, A8806 adjusted to pH 7.4) to obtain a 5 mM palmitate-BSA stock solution that was filtered using a 0.22-μm low-protein binding filter (Millipore). BSA/ethanol was used in control treatments during the protocol. In some experiments during the 24 h metabolic activation BMDMs were treated with PP2 (Sigma Aldrich, 529573), a reversible ATP-competitive inhibitor of the Src family of protein tyrosine kinases, in 2 µM final concentration or 0.5 mg/ml RGD peptide (Cayman Chemical, 529573).

### Cell culture and differentiation of 3T3 cells

3T3-L1 murine preadipocytes (ATCC:CL-173) were maintained as subconfluent cultures in Dulbecco’s modified Eagle’s medium (DMEM, Sigma-Aldrich) supplemented with 4.5 g/l D-glucose, 2 mM L-glutamine and 10% (v/v) bovine calf serum. For differentiation, after 2 days post-confluency, differentiation was induced by addition of 2 μg/ml insulin, 0.5 mM isobutylmethylxanthine (Sigma-Aldrich, I5879), and 0.25 μM dexamethasone (Sigma-Aldrich, D4902). The cells were maintained in this medium for 3 days and then for 2 more days in medium supplemented with 1 μg/ml insulin. After 10 days of differentiation, FBS was omitted from the medium and the cells were maintained for another day in order to induce apoptosis^55^. The basal cell death index was very low (<5%), but following serum withdrawal the percentage of annexin V positive cells increased significantly (≥90 after 24 h).

### Insulin resistance test, intraperitoneal glucose tolerance test (IPGTT), insulin determination

Insulin resistance test and IPGTT was performed on week 15 and 16, respectively. After a 6 h fasting period, 0.75 IU/bwkg insulin (ACTRAPID Penfill 100 IU/ml, Novo Nordisk) or 2 g/bwkg glucose was injected intraperitoneally. Blood glucose levels were determined with DCont Trend glucose monitor (DCont, Hungary) at the indicated time points after insulin or glucose injection. Plasma insulin levels were determined by Mouse Insulin ELISA kit (ALPCO, 80-INSMS-E01) according to the manufacturer's instructions.

### Collection of tissue samples

At the end of the feeding phase, the animals were sacrificed with isoflurane, body weight, body length, liver, and gonadal white adipose tissue (gWAT) weight were measured. Blood, liver, and gWATsamples were collected for subsequent analysis. Blood samples were allowed to clot and spun at 12.000 rpm for 15 min to collect plasma. For histological analysis, adipose tissue and liver samples were fixed in 4% paraformaldehyde; for gene expression and liver triglyceride determination were frozen in liquid nitrogen and stored at −80 °C prior to extraction.

### Adipocyte and adipose tissue macrophage (ATM) isolation

gWAT tissue was dissected and washed and kept in transport buffer (DMEM supplemented with 1% Penicillin–Streptomycin solution and 1% bovine serum albumin (BSA). The tissue was minced, digested for 30–60 min at 37 °C in digestion solution (HEPES buffer (pH 7.4, H3375) supplemented with 20 g/L BSA and 0.5 g/L collagenase type 1 (C6885); all from Sigma-Aldrich, and filtered through a nylon filter (100 μm). After a centrifugation step, adipocytes were collected and stored at −80 °C prior to RNA isolation. After the removal of red blood cells by hemolysis (ACK Lysing Buffer, Thermo Fisher Scientific, A1049201) the stromal vascular cell fraction was resuspended in staining buffer (PBS supplemented with 0.5% BSA and 2 mM EDTA (Sigma-Aldrich, E6758)). ATMs were isolated from SVCs using MACS Technology (F4/80-based positive selection; (Miltenyi Biotech, 130-097-050, 130-117-509) according to the manufacturer’s instructions.

### Liver triglyceride levels

Triglyceride concentrations were quantified in saponified, neutralized liver extract (digested in ethanolic KOH (Sigma-Aldrich, P5958) overnight at 55 °C by glycerol enzymatic assay (Free Glycerol Reagent, Glycerol Standard Solution, Sigma-Aldrich, F6428, G7793) according to the manufacturer's instructions.

### Histology and immunohistochemistry

Hepatic tissues were fixed in 4% neutral buffered formaldehyde (Sigma-Aldrich, F8775) and embedded in paraffin. Paraffin sections were stained with hematoxylin-eosin (H&E; Sigma-Aldrich, HT110116, GHS116) stain. Histological sections were analyzed on Leica DMRB/E light microscope (Heerbrugg, Switzerland).

After paraffin embedding, 6 µm thick sections were cut from gWAT tissue samples. The sections were mounted on glass slides, deparaffinized and kept in 10% normal goat serum (Thermo Fisher Scientific, 31872) for 50 min at room temperature. The sections were first incubated with an antibody raised against the macrophage marker F4/80 in rat (diluted 1:1000, Hycult Biotech, HM1066), then transferred into a solution of goat anti-rat IgG conjugated with Alexa Fluor 555 (diluted 1:1000, Molecular Probes, A21434). We have noticed that the fluorescein-labeled anti-digoxigenin antibody offered by the Apoptag fluorescein kit (Millipore, S7110) has strong aspecific binding in our adipocyte tissue sections. This aspecific signal was used to visualize the cellular architecture of gWAT tissue. The immunostained sections were covered with a Vectashield Antifade Mounting Medium with DAPI (Vector Laboratories, H-1200). The number of adipocytes and nuclei was determined by ImageJ software, while the number of CLS was counted manually.

### Confocal microscopy

Single 1-μm-thick optical sections were scanned with an Olympus FV3000 confocal microscope. Scanning was carried out with a ×40 oil-immersion lens (NA 1.3). The confocal settings (laser power, confocal aperture, and gain) were identical for all means, and care was taken to ensure that no pixels corresponding to puncta immunostained for the F4/80 macrophage marker were saturated. The scanned images were processed by Adobe Photoshop CS3 software and analyzed by ImageJ software.

### RNA Isolation and real-time quantitative (RT-qPCR)

TRIzol reagent (UD-Genomed, URN0102) was used to isolate total RNA from samples. cDNA was synthesized with High-Capacity cDNA Archive Kit (Thermo Fisher Scientific, 4368813) according to the manufacturer's instruction. Gene expression levels were determined with qRT-PCR using FAM-MGB labeled Taq-Man probes (Thermo Fisher Scientific) by Real-time PCR on Roche Light Cycler 480 platform. Samples were run in triplicates. Expression values were normalized to GAPDH housekeeping gene.

### Time-lapse imaging microscopy

3T3-L1 originated adipocytes were stained with Nile red to identify lipid droplets, while macrophages with Hoechst 33342 (Thermo Fischer Scientific, 62249) to recognize macrophage nuclei and with 10 μM 5-carboxyfluorescein diacetate (CFDA) (Sigma-Aldrich, C4916) to recognize macrophage cytosol. Wild type or TG2 null macrophages were layered on top of the apoptotic adipocytes in a ratio of 5:1. The co-culture was placed in a temperature-, humidity- and CO_2_-controlled, motorized Olympus IX-81 inverted microscope (Olympus America), which was equipped with a cooled Hamamatsu ORCA-R2 (Hamamatsu Photonics, Hamamatsu City, Japan) high-resolution monochrome CCD camera and a DP21-CU 2-megapixel digital color camera (Olympus America). Cells were monitored for 5 h and in every 5 min, an image was taken. Data were converted into a video file with the use of the Xcellence software (Olympus America). Lipid contents of adipocytes induced to undergo apoptosis by serum starvation were labeled by Nile red (Thermo Fisher Scientific, N1142).

### Western blot

Mme BMDMs were washed with PBS, dissociated by lysis buffer (50 mM Tris (T5941) pH 6.8, 2% SDS (L3771), 5% glycerol (G5516), 2 mM DTT (D0632), 2.5 mM EDTA, 2.5 mM EGTA (E3889), all reagents from Sigma-Aldrich), supplemented protease and phosphatase inhibitor cocktail (Sigma-Aldrich, P8340, 524629). Samples were boiled for 10 min with SDS-sample buffer at 100 °C. The samples were separated by 12% SDS-PAGE and transferred to PVDF membrane (Bio-Rad, 1620177). The membrane was blocked with TBST buffer (10 mM Tris, pH 8.0, 0.15 M NaCl (S7653), and 0.05% Tween 20 (P1379); all reagents from Sigma-Aldrich) containing 5% BSA for 1 h at room temperature and then probed with anti-phospho(Tyr416)-Src, c-Src (Cells Signaling Technology, 2101, 2108) and beta-actin (Sigma-Aldrich, 5441) primary antibodies overnight at 4 °C. Beta-actin was used for the loading control. After washing with TBST buffer, blots were incubated with HRP-conjugated secondary antibodies for 1 h at room temperature. Proteins were detected with chemiluminescence (Immobilon Western Chemiluminescent HRP Substrate, Merck Millipore, WBKLS0500). The pixel density of bands was determined by Image J software.

### Statistical analysis

Data are presented as mean ± SD for all data. All statistical analyses were performed using GraphPad Prism 6.01 and a *P*-value < 0.05 was considered as significant and is indicated by asterisk (*). For differences between 2 groups 2-tailed unpaired Student’s *t*-test, for comparisons *n* > 2 groups one- ANOVA (with Turkey’s multiple comparisons test) was used. *n* = 8 mice/group were used for the experiments except for BM transplanted population, where *n* = 5 mice were investigated. For datasets split on two independent factors, 2-way ANOVA was used.

## Supplementary information

Supplementary Figure S1

Supplementary Figure 2

Supplementary Figure 3

Supplementary Figure 4

Legends to the supplementary videos

Supplementary Video 1

Supplementary Video 2

Supplementary Video 3

Supplementary Video 4
