# Supplementary material for: Loss of transglutaminase 2 sensitizes for diet-induced obesity-related inflammation and insulin resistance due to enhanced macrophage c-Src signaling
Source: Cell Death Dis. 2019 Jun 5;10(6):439. doi: 10.1038/s41419-019-1677-z (PMC6549190; doi:10.1038/s41419-019-1677-z)
Supplement: Supplementary file 1 — Supplementary Figure S1 [file 41419_2019_1677_MOESM1_ESM.docx]

**Supplementary Figure S1: Enhanced adipocyte apoptosis in the gonadal fat during diet induced obesity in TG2^-/-^ mice.**

**(a-f**) Relative gene expression levels of apoptosis related Bid, Bim, Bcl-2, Bcl-XL, Mcl-1 and TG2 in gonadal fat adipocytes of wild type and TG2 null mice fed on ND, HSD or HFD determined by qRT-PCR at the end of the feeding period. GAPDH was used as a reference gene. Results are expressed as mean ± SD (n = 8 mice per group). Statistical significance was evaluated by one-way ANOVA test (*p<0.05). (**g**) Paraffin-embedded gWAT slides from wild type and TG2 null mice fed on various diets were stained with the non-specifically labeling anti-digoxin antibody, anti-F4/80 antibody and DAPI to visualize adipocytes, macrophages and nuclei under confocal microscopy. Scale bar, 100 μm. CLS cells in fields from randomly selected sections of three different mice in each group were quantified. Results are expressed as mean ± SD (n = 3 mice per group). Statistical significance was evaluated by 2-tailed unpaired Student’s t-test (*p<0.05).

**Supplementary Figure S2: Enhanced hepatic steatosis, insulin resistance and fasting serum insulin levels during diet-induced obesity in TG2^-/-^ mice.**

**(a**) Liver weights of wild type and TG2 null mice fed on ND, HSD or HFD at the end of the feeding period. (**b**) Liver triacylglycerol content of wild type and TG2 null mice fed on ND, HSD or HFD at the end of the feeding period. Triacylglycerol content was determined from saponified, neutralized liver extracts by glycerol enzymatic assay. (**c**) Paraffin-embedded liver tissue slides from the same mice were stained with H&E to visualize tissue architecture. One representative series of three are shown. Scale bar, 250 μm. (**d**) Intraperitoneal glucose tolerance test results of wild type and TG2 null mice fed on ND, HSD and HFD diets. (**e)** Insulin resistance values of wild type and TG2 null mice kept on ND, HSD or HFD from the test performed on the 15th week of the feeding period. (**f**) Serum insulin levels of wild type and TG2 null mice kept on ND, HSD or HFD at the end of the feeding period determined by Mouse Insulin ELISA kit. Data are presented as mean±SD (n = 8 mice per group). Statistical significance was evaluated by one-way ANOVA (*p<0.05).

**Supplementary Figure S3: Loss of TG2 in non-BMD cells sensitizes for insulin resistance in mice fed on HFD.**

(**a**) Weekly body weight gains on HFD of TG2^+/+^, TG2^-/-^ mice, of TG2^+/+^ and TG2^-/-^ mice transplanted with bone marrow from BoyJ mice and of BoyJ mice transplanted with bone marrow from TG2^+/+^ or TG2 ^-/-^ mice. (**b**) Confocal images of gWAT collected from TG2^+/+^ and TG2^-/-^ mice transplanted with the bone marrow of BoyJ mice at the end of HFD feeding period. Paraffin-embedded gWAT slides were stained with the non-specifically labeling anti-digoxin antibody, anti-F4/80 antibody and DAPI to visualize adipocytes, macrophages and nuclei under confocal microscopy. Scale bar, 100 μm. CLS cells in fields from randomly selected sections of three different mice in each group were quantified. Results are expressed as mean ± SD (n = 3 mice per group). Statistical significance was evaluated by 2-tailed unpaired Student’s t-test (*p<0.05). (**c**) Weights of gWAT from the mice described in *b*. (**d**) Relative gene expression levels of apoptosis related Bid in gWAT adipocytes from the same mice. **e** Liver weights from the same mice. (**f**) Liver triacylglycerol contents from the same mice determined from saponified, neutralized liver extracts by glycerol enzymatic assay. (**g**) Paraffin-embedded liver tissue slides from the same mice stained with H&E to visualize tissue architecture. One representative series of three are shown. Scale bar, 250 μm. (**h**) Relative gene expression levels of adipokine in gWAT adipocytes from the same mice. (**i**) Inflammatory cytokine and resistin relative gene expression levels of gWAT macrophages from the same mice determined by qRT-PCR using GAPDH as a reference gene. (**j**) Insulin resistance values of the same mice. Insulin resistance test was performed on week 15 (6 hr fasting followed by intraperitoneal administration of 0.75 IU/bwkg insulin). (**k**) Serum insulin levels determined by Mouse Insulin ELISA kit. Data are presented as mean±SD (n = 8 mice per group). Statistical significance was evaluated by one-way ANOVA (*p<0.05).
