# Supplementary material for: Loss of transglutaminase 2 sensitizes for diet-induced obesity-related inflammation and insulin resistance due to enhanced macrophage c-Src signaling
Source: Cell Death Dis. 2019 Jun 5;10(6):439. doi: 10.1038/s41419-019-1677-z (PMC6549190; doi:10.1038/s41419-019-1677-z)
Supplement: Supplementary file 5 — Legends to the supplementary videos [file 41419_2019_1677_MOESM5_ESM.docx]

**LEGENDS TO THE SUPPLEMENRARY VIDEOS**

**Supplementary video 1**

One apoptotic adipocyte and one macrophage pair demonstrating the process of lysosomal exocytosis by wild type macrophages. 3T3 adipocytes induced to die by serum withdrawal for 24 h were stained for lipids with Nile red and exposed to wild type BMDMs stained with vital Hoechst DNA dye for nuclei (blue). Apoptotic adipocyte clearance was followed for 5 hours by laser scanning microscopy. Macrophages without lipid uptake appear as a blue nucleus. Macrophages taking up lipids appear with a blue nucleus and a red cytosol. Please note a continuous apoptotic adipocyte-derived lipid containing vesicle uptake by the wild type macrophage.

**Supplementary video 2**

Clearance of several apoptotic adipocytes by wild type BMDMs. 3T3 adipocytes induced to die by serum withdrawal for 24 h were stained for lipids with Nile red (red) and exposed to wild type BMDMs stained with vital Hoechst DNA dye for nuclei (blue). Apoptotic adipocyte clearance was followed for 5 hours by laser scanning microscopy. Macrophages without lipid uptake appear as a blue nucleus. Macrophages taking up lipids appear with blue nucleus and red cytosol. Please note the continuous lipid containing vesicles’ uptake by many wild type macrophages and slow or no shrinkage of the adipocytes.

**Supplementary video 3**

One apoptotic adipocyte and TG2 null macrophage pair demonstrating the process of lysosomal exocytosis and clearance of apoptotic adipocytes by TG2 null macrophages. 3T3 adipocytes induced to die by serum withdrawal for 24 h were stained for lipids with Nile red and exposed to wild type BMDMs stained with vital Hoechst DNA dye for nuclei (blue). Apoptotic adipocyte clearance was followed for 5 hours by laser scanning microscopy. Macrophages without lipid uptake appear as a blue nucleus. Macrophages taking up lipids appear with blue nucleus and red cytosol. Please note the fast lipid containing vesicles’ uptake by the TG2 null macrophage followed by shrinkage and membrane blebbing of the targeted apoptotic adipocyte.

**Supplementary video 4**

Clearance of several apoptotic adipocytes by TG2 null BMDMs. 3T3 adipocytes induced to die by serum withdrawal for 24 h were stained for lipids with Nile red (red) and exposed to TG2 null BMDMs stained with vital Hoechst DNA dye for nuclei (blue). Apoptotic adipocyte clearance was followed for 5 hours by laser scanning microscopy. Macrophages without lipid uptake appear as a blue nucleus. Macrophages taking up lipids appear with blue nucleus and red cytosol. Please note the fast lipid containing vesicles’ uptake by a number of TG2 null macrophages followed by shrinkage and membrane blebbing of the targeted apoptotic adipocytes.
